# Gharial ( *Gavialis gangeticus* , Gmelin 1789) Beyond Protected Areas of Nepal: Population, Reproduction, and Seasonal Migration of Gharial in the Karnali River and Its Tributaries

**DOI:** 10.1002/ece3.74133

**Published:** 2026-08-04

**Authors:** Ashish Bashyal, Nischal Shrestha, Rosy Thapa, Bhojraj Dhungana, Ashok Kumar Ram, Sabanam Pathak, Sandeep Shrestha

**Affiliations:** ^1^ Biodiversity Conservancy Nepal Rupandehi Nepal; ^2^ Dolphin, Aquatic and Biodiversity Conservation Nepal Kailali Nepal; ^3^ Department of National Parks and Wildlife Conservation Kathmandu Nepal; ^4^ Department of Forests and Soil Conservation Kathmandu Nepal

**Keywords:** Bardiya, crocodile, dolphin, Ghaghara River, nest, non‐protected

## Abstract

Habitats beyond protected areas are essential for crocodilian conservation, as many species utilize these extended habitats for basking, nesting, foraging, and seasonal movements. Gharial ( *Gavialis gangeticus* ) is a Critically Endangered apex predator in freshwater ecosystems whose populations in Nepal are restricted primarily within or adjacent to protected areas, with few records from the south‐western lowlands. In this study, we gained insights into the status of the gharial population residing in the Karnali River and its tributaries—the Mohana, Patthariya, and Kaande—by providing the first systematic accounts of their counts, nesting and reproduction, and possible seasonal migration. We conducted surveys before, during, and after the monsoons in 2024 and 2025 in 76 km of these river reaches. We recorded the highest counts for gharials for all rivers pooled during monsoon surveys in 2024 (*n* = 8) and 2025 (*n* = 12), and the lowest during post‐monsoon surveys in 2024 (*n* = 4) and 2025 (*n* = 3). Gharial number ranged between one and seven in the Karnali and between zero and five each in the Mohana and the Patthariya across surveys in both years. The gharial population is dominated by adult females with no adult males recorded. We documented a total of 16 gharial nests containing 427 live hatchlings from 509 eggs with a hatching success of over 80%, including both years. Our results showed that there is a small resident gharial population residing in the Karnali River system, and they are reproducing successfully. There is likely seasonal movement of gharials among the Karnali and its tributaries, and possible transboundary movement along the Karnali between Nepal and India. We underscore the need for protecting these riverine habitats of gharials beyond protected areas and for coordinated transboundary research and conservation efforts to ensure long‐term gharial conservation.

## Graphical abstract

Populations of Critically Endangered gharial ( *Gavialis gangeticus* ) in Nepal are restricted primarily within or adjacent to protected areas. There are few records of gharial occurrence from the Karnali River and its tributaries outside protected areas in southwestern Nepal but there is very little information available for gharials residing in the region. In this study, we gained insights into the status of the gharial population residing in the Karnali River system by providing the first systematic accounts of their counts, nesting and reproduction, and seasonal migration.

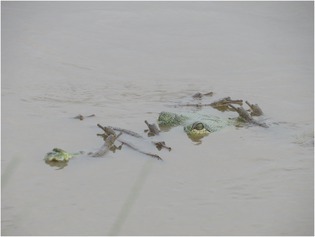

## 1 Introduction

Protected and conserved areas cover 17.54% of the global terrestrial and inland water surface and 8.45% of marine areas currently and are central to global efforts in protecting and maintaining biodiversity (UNEP‐WCMC and IUCN 2024). Protected areas provide safe havens allowing wildlife populations to thrive by reducing anthropogenic stressors and maintaining key ecological processes. Networks of protected and conserved areas keep expanding globally, but these networks are not well connected (UNEP‐WCMC and IUCN 2024). Protected areas play an important role in protecting biodiversity globally; nonetheless, their effectiveness is closely linked to ecological conditions and management of adjacent habitat beyond protected areas (DeFries et al. 2010). These non‐protected areas often function as corridors or supplementary habitats, thereby enhancing connectivity between protected areas or populations, especially for wide‐ranging or aquatic species with larger home ranges (Margules and Pressey 2000; Kremen et al. 2007).

Crocodilians are one of the largest inhabitants of freshwater ecosystems and are known to traverse incredibly long distances (Campbell et al. 2010; Mascarenhas‐Junior et al. 2023). There are 26 species of crocodilians currently recognized, and among them, seven are Critically Endangered (Vliet et al. 2024). Two‐thirds of crocodilian species globally are represented in protected area networks, albeit with varying levels of protection and management regimes (Lourenço‐de‐Moraes et al. 2023). As such, protected areas are crucial for the conservation of many threatened crocodilians (Stevenson et al. 2025; Lourenço‐de‐Moraes et al. 2023). Nonetheless, the river reaches outside the protected areas also play important roles in crocodilian conservation as many species use extended habitat far beyond the boundaries of protected areas for basking, nesting, foraging, and seasonal movement (Rainwater et al. 2019; Stevenson et al. 2025). These habitats also act as crucial corridors providing connectivity among populations and facilitating dispersal and gene flow. However, the ecological roles of these habitats outside protected areas in the survival and well‐being of crocodilians are often overlooked (Rainwater et al. 2019; Stevenson et al. 2025; Lourenço‐de‐Moraes et al. 2023).

The Gharial is an apex predator in freshwater ecosystems identified by its long, tubular snout, likely adapted for catching fish (Stevenson and Whitaker 2010). It is classified as “Critically Endangered” in the IUCN Red List and protected by law in Nepal and India (Lang et al. 2019). Historically, gharials were common in major river systems and their tributaries in the Indian subcontinent (Stevenson and Whitaker 2010). Gharials experienced severe declines in their historic population size (from > 20,000 to 650 adults) and in the extent of their range (from > 40,000 to 5000 km^2^) globally, primarily owing to threats such as habitat loss and alteration, including construction of dams, unregulated hunting and removal of eggs, and accidental drowning in fishing nets (Stevenson and Whitaker 2010). Gharials are presently confined to 14 widely spaced locations in northern India and lowland Nepal (Lang et al. 2019). Only six out of these 14 sub‐populations have exhibited recent reproduction/recruitment, the majority of which occur within or adjacent to protected areas (Lang et al. 2019; Bashyal et al. 2019, 2024).

In the lowlands of Nepal, gharial populations are restricted primarily within or adjacent to protected areas, including the Chitwan and Bardiya National Parks (Bashyal et al. 2021). Consequently, research and conservation efforts such as ex‐situ breeding and captive‐rearing, population reinforcements by releasing captive‐reared gharials, population surveys, and local capacity building have been confined primarily within these protected areas (Khadka and Bashyal 2019; Bashyal et al. 2021; Pathak et al. 2023; Khadka et al. 2020, 2024). Additionally, there is a scant record of the occurrence of a small number of gharials from the Karnali River and its tributaries outside the Bardiya National Park (BNP), near the Indo‐Nepal border (DNPWC 2018).

Given the small population size and highly restricted range of gharial, it is crucial to identify populations outside protected areas, regardless of their size, and to have updated information on various ecological attributes of their population. Long‐term conservation of gharial can't be realized without the availability of robust information (Stevenson and Whitaker 2010; DNPWC 2018). In this study, we gained insights into the status of the gharial population residing in the Karnali River and its tributaries—the Mohana, Patthariya, and Kaande beyond protected areas by addressing the following questions: (a) What is the size, structure, and sex ratio of the gharial population residing in the Karnali and its tributaries? (b) Are gharials reproducing in these rivers, and if so, what is the status of hatchling survival and recruitment? and (c) Is there possibly a seasonal migration of gharials in the Karnali and its tributaries?

## 2 Materials and Methods

### 2.1 Study Area

**FIGURE 1:**
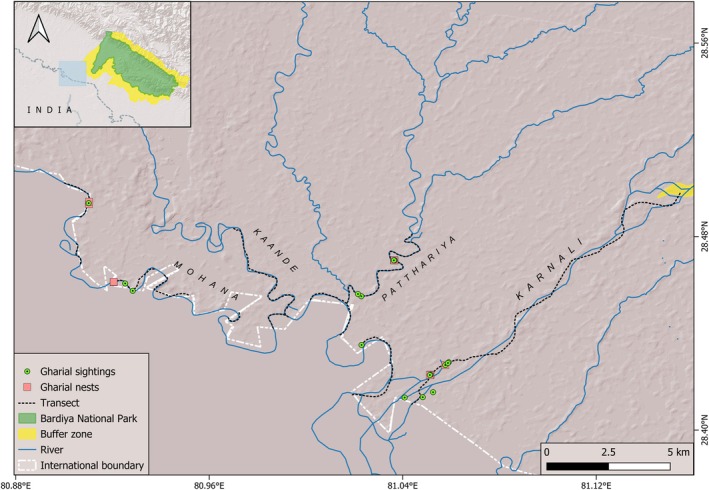
Map of study area showing the survey reaches of the Karnali River and its tributaries, including the Mohana, Patthariya, and Kaande Rivers (black dashed line), indicating locations of gharial sightings (green circle) and nests (pink square).

The Karnali is a snow‐fed and perennial river originating from the Tibetan Plateau. The Karnali enters the BNP on its north‐western corner at the Chisapani Bridge and bifurcates into two major channels flowing south. The western channel of the Karnali flows outside the BNP, whereas the eastern channel (referred to as Geruwa) flows mostly through the BNP (Figure 1; Bashyal et al. 2021). The western channel (Kaudiyala in India) and the eastern channel (Girwa in India) merge ~20 km downstream from the Indo‐Nepal border in Girijapuri barrage to form the Ghaghara River (Vashistha et al. 2024, 2025). There is a resident gharial population in the Kaudiyala and Girwa Rivers in and around the Katerniaghat Wildlife Sanctuary in India (Vashistha et al. 2025).

Mohana and its tributaries—the Kaande and the Patthariya—are warm‐water systems originating in the Churia hills of western Nepal. The Mohana River meanders between Nepal and India multiple times, finally flowing into the Karnali (Figure 1). Patthariya and Kaande are smaller tributaries of Mohana. These rivers have low flow during dry seasons. This study was conducted in the stretch of the Karnali River outside the jurisdiction of the BNP (western channel), and its tributaries—the Kaande, Patthariya, and Mohana Rivers (Figure 1).

### 2.2 Population Assessment

**FIGURE 2:**
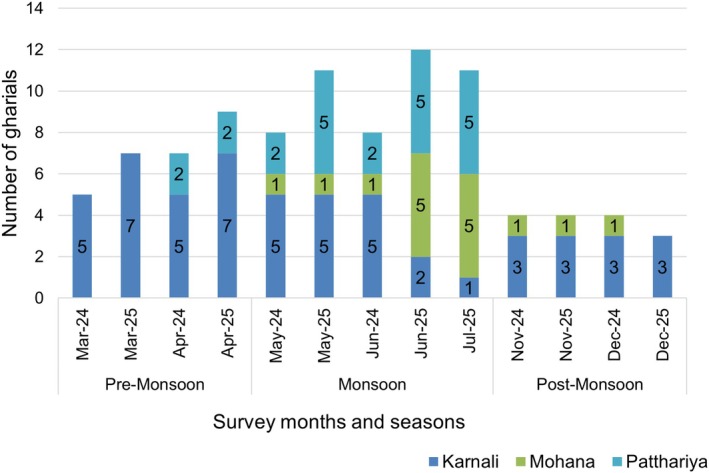
Number of gharials observed in the Karnali, Mohana, and Patthariya in pre‐monsoon, monsoon, and post‐monsoon months in 2024 and 2025.

**FIGURE 3:**
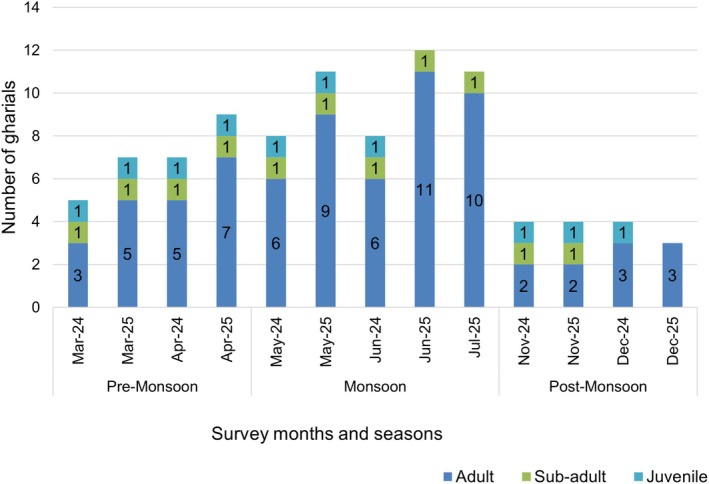
Size‐class categories of the gharials observed in the Karnali, Mohana, and Patthariya in pre‐monsoon, monsoon, and post‐monsoon months in 2024 and 2025.

We conducted daylight surveys (0900–1600) in a rubber boat or on foot for a total of 76 km river reaches including the Karnali (40 km) and its tributaries—Patthariya (8 km), Kaande (6.5 km), and Mohana (21.5 km; Figure 1). Since the Mohana meanders between Nepal and India multiple times, we surveyed only the river reaches within the Nepalese territory (Figure 1). In 2024 and 2025, we surveyed these rivers 13 times to count gharials during pre‐monsoon (February–April), monsoon (May–July) and post‐monsoon (November–December; Figures 2 and 3). We conducted boat surveys in the Karnali River since we had to survey longer reach (40 km) and water level was high in all seasons. Boat surveys involved two individuals paddling rubber boat moving at an average speed of ~6 km/h and two surveyors scanning riverbanks with binoculars simultaneously. Similarly, we conducted foot surveys in the Mohana, Patthariya, and Kaande due to low water levels, relatively shorter survey reaches, and jurisdictional limitations preventing boat surveys in Indian territory of the Mohana. Foot surveys involved two to four surveyors walking along the riverbanks with an average speed of ~3.5 km/h and briefly pausing at every 100–200 m to scan riverbanks with binoculars.

For gharials basking with full body visible, we estimated their approximate “Total Length” (TL; distance from the anterior tip of the snout to the posterior tip of the tail) by visual examination with binoculars or naked eye, based on our years of experience in gharial surveys. For gharials swimming with only their head visible, we estimated the approximate length of their head and multiplied it by 6.5 to calculate their TL (Whitaker and Whitaker 2008). Based on the approximate TL, we classified gharials as hatchlings (≤ 1 m TL); juveniles (> 1–2 m TL), sub‐adults (> 2–3 m TL), adult females (3–4 m TL) and adult males (> 4 m TL) with “ghara”—a protuberance at the tip of snout (Lang and Kumar 2016; Lang et al. 2018). Classifying gharials into various size‐categories by estimating their TL by visual observation by experienced surveyors is commonly used method across the species' range (Lang et al. 2019; Bashyal et al. 2021; Vashistha et al. 2025). We took photographs of each gharial and recorded GPS locations using a geographic coordinate system (decimal degrees) in the World Geodetic System 1984 (WGS 84) datum with a hand‐held GPS device (Garmin GPSmap64). We opted for two survey techniques (boat and on foot surveys) due to logistical limitations owing to length of river reaches surveyed, water level and jurisdictional limitations and therefore gharial counts for both methods were combined. Survey efforts were not equal among rivers due to differences in length of river reaches surveyed and survey techniques.

### 2.3 Reproductive Ecology

During the nesting season of gharials in March–April 2024 and 2025, we surveyed the Karnali and its tributaries to document gharial nests (Lang and Kumar 2016; Khadka et al. 2020). We scanned the riverbanks for any signs of nesting activities such as the presence of trial nests (gharials build trial nests before laying the eggs to check for the substrate of nesting areas), body prints, entry and exit trails to and from water's edge, as well as disturbances in the sand (Khadka et al. 2020; Bashyal et al. 2024). We also observed behaviors of adult female gharials such as regular movement to certain spots to and from the water's edge and exhibition of defensive behavior during the movement of humans/livestock near the potential nest to further confirm the evidence of nesting. These behaviors have been documented in nesting female gharials across India and Nepal (Lang and Kumar 2016; Khadka et al. 2020; Bashyal et al. 2024).

During the hatching season in June–July 2024 and 2025, we documented evidence of hatchlings, including excavated nests, live hatchlings, and remnant eggshells. We revisited nesting sites every day from 10 June until the eggs hatched in both years. When gharial eggs are ready to hatch, hatchlings make high‐pitched calls signaling their mother, who will then open the nest. Hatchlings use their egg tooth to crack the eggshells and move quickly towards water themselves as mother gharials do not help transport hatchlings due to their unique slender snout (Lang et al. 2019). When nesting gharials excavated the nest, we counted the total number of eggs that hatched and eggs that did not hatch to estimate the clutch size. Similarly, we counted gharial hatchlings basking by the riverbank and/or swimming to estimate the total number of hatchlings per nest. In some instances, when hatchlings from nearby nests had aggregated to form a crèche, as gharials are known to nest and guard hatchlings communally (Lang et al. 2019), we counted the total number of hatchlings in that crèche. We calculated hatching success by dividing the total number of live hatchlings by the clutch size. We also recorded the distance and height of the nest from the riverbank, and the aspect of the nest since nest selection by gharials is likely influenced by these variables, as reported from Bardiya and Chitwan National Parks (Khadka et al. 2020; Bashyal et al. 2024).

### 2.4 Data Analysis

We conducted descriptive statistical analysis in R (R Core Team 2025) employing package “readxl” (Wickham et al. 2023) and presented mean values as ±1 SD. We designed a map in QGIS 3.40 (QGIS Development Team 2025).

## 3 Results

### 3.1 Gharial Counts in the Karnali and Its Tributaries

In 2024, we recorded the highest gharial count pooled for all four rivers during the monsoon surveys in May and June (*n* = 8 in both months), which consisted of five gharials in the Karnali, one in Mohana and two in Patthariya (Figures 1 and 2). Similarly, in 2025, we recorded the highest gharial count during the monsoon survey in June (*n* = 12) which consisted of two in the Karnali, and five each in the Mohana and the Patthariya Rivers (Figure 2). We recorded the lowest gharial count pooled for all four rivers during post‐monsoon surveys in November and December in both 2024 and 2025. Gharial count ranged between one and seven in the Karnali and between zero and five each in the Mohana and the Patthariya across surveys in both years (Figure 2). We did not observe any gharials in the Kaande River in any of our surveys. The gharial population in the Karnali and its tributaries was dominated by the adult females, as six out of a maximum of eight gharials recorded in 2024 and 11 out of a maximum of 12 gharials recorded in 2025 were adult females (Figure 3). Only one juvenile and one sub‐adult gharial were recorded in both years (Figure 3). No adult males were observed.

### 3.2 Nesting and Reproduction in the Karnali and Its Tributaries

**FIGURE 4:**
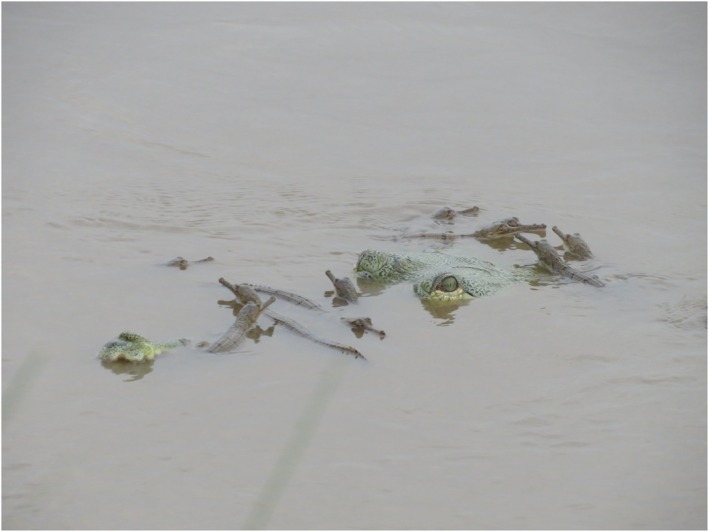
Newly hatched gharial hatchlings along with their mother gharial in the Patthariya River in June 2024. Photo credit: Ashish Bashyal.

During the nesting season in both years, we observed abundant signs in the Karnali and its tributaries except the Kaande. Signs included trial nests, entry and exit trails to and from the water's edge, body prints, and disturbances in the sand. During the hatching season in 2024, we recorded a total of five gharial nests including three in the Karnali and two in the Patthariya Rivers (Table 1; Figure 1). Altogether, these five nests contained 178 eggs (mean = 35.6), out of which 128 eggs hatched successfully (mean = 25.6), resulting in hatching success of over 70% (Table 1; Figure 4). In 2025, we recorded a total of 11 gharial nests including one in the Karnali, five each in the Patthariya and the Mohana Rivers. These nests contained a total of 331 eggs (mean = 30.09), of which 299 eggs hatched successfully (mean = 27.18), resulting in hatching success of 90.3% (Table 1; Figure 1).

**TABLE 1:** Description of ecological and clutch characteristics of gharial nests in the Karnali River and its tributaries in June 2024 and 2025.

Year	Nest	River	Hatching date	Distance of nest from riverbank (m)	Height of the nest from the riverbank (ft)	Aspect of the nest	Clutch size	Live hatchlings	Hatching success (%)
2024	1	Patthariya	2024‐06‐19	8.4	10	188 S	34	24	70.59
2024	2	Patthariya	2024‐06‐18	6.5	7.5	170 S	25	20	80.00
2024	3	Karnali	2024‐06‐18	4.1	5	125 SE	40	33	82.50
2024	4	Karnali	2024‐06‐23	7	6.5	148 SE	40	40	100
2024	5	Karnali	2024‐06‐25	5.3	3.7	149 SE	39	11	28.21
2025	6*	Patthariya	2025‐06‐15	9	5	192 S	94	86	91.49
2025	7*	Patthariya	2025‐06‐15	8	8.9	186 S			
2025	8*	Patthariya	2025‐06‐15	5.4	7.2	182 S			
2025	9*	Patthariya	2025‐06‐15	5.7	8	190 S			
2025	10	Patthariya	2025‐06‐21	5.8	7	187 S	37	37	100.00
2025	11*	Mohana	2025‐06‐18	2.19	4.5	137 SE	80	79	98.75
2025	12*	Mohana	2025‐06‐18	2	5	127 SE			
2025	13	Mohana	2025‐06‐26	2.2	5.5	170 S	31	29	100
2025	14	Mohana	2025‐06‐18	8	9.5	274 W	34	33	97.06
2025	15	Mohana	2025‐06‐20	6.7	8.3	267 W	17	0	0.00
2025	16	Karnali	2025‐06‐25	3.2	3.8	137 SE	38	35	92.11
	2024 mean			6.26 ± 1.47	6.54 ± 2.16		35.6	25.6	71.91
	2024 sub‐total						178	128	
	2025 mean			5.29 ± 2.44	6.61 ± 1.85		30.09	27.18	90.33
	2025 sub‐total						331	299	
	Patthariya mean			6.97 ± 1.36	7.66 ± 1.46		27.14	23.86	87.89
	Patthariya sub‐total						190	167	
	Mohana mean			4.22 ± 2.59	6.56 ± 1.97		32.40	28.20	87.04
	Mohana sub‐total						162	141	
	Karnali mean			4.90 ± 1.42	4.75 ± 1.13		39.25	29.75	75.80
	Karnali sub‐total						157	119	
	Overall mean			5.59 ± 2.23	6.59 ± 1.95		31.81	26.69	83.89
	Grand total						509	427	

* Nests 6–9 in the Patthariya and nests 11 and 12 in the Mohana had already formed creches each when we first documented these nests, and thus their data is presented as respective groups.

Overall, we recorded a total of 16 gharial nests containing 427 live hatchlings from 509 eggs with a hatching success of over 80% including both the years (Table 1; Figure 1). On average, nests contained ~31 eggs resulting in ~26 hatchlings per nest (Table 1; Figure 4). The mean clutch size was higher in 2024, but the mean number of live hatchlings and the hatching success were higher in 2025. Similarly, the mean clutch size and the mean number of live hatchlings were higher in the Karnali, but the hatching success was higher in the Patthariya (Table 1). On average, nests were 5.59 m away and 6.59 ft higher from the riverbank (Table 1). Nests were farther from the riverbank in 2024 but were higher from the riverbank in 2025. Similarly, nests were farther and higher from the riverbank in the Patthariya (Table 1). Gharial nests were facing South, Southeast, or West, indicating that nests were strategically oriented to receive higher solar radiation. All nests hatched during a narrow window between 15 and 26 June in both years (Table 1).

Among the four rivers surveyed in this study, we recorded gharial nests from the Karnali and the Patthariya in both years, whereas we recorded nests from the Mohana only in 2025, and no nests were recorded from Kaande (Table 1; Figure 1). Overall, we recorded the highest number of nests from the Patthariya (*n* = 7) and the lowest from the Karnali (*n* = 4; Table 1). We recorded all the nests in Patthariya at the same nesting site, with nests spreading a few meters from each other in both years. In the Mohana, we recorded nests from two nesting sites, and in the Karnali, nests were recorded within 2 km of each other.

## 4 Discussion

### 4.1 Status of Gharials in the Karnali and Its Tributaries

There is scant information available for gharials in the Karnali River, and most of that available information is from the eastern channel, which flows mostly through the BNP (Lang et al. 2019; Bashyal et al. 2021). Discounting some preliminary surveys in the upper stretch of the western channel (Bashyal et al. 2021), to the best of our knowledge, there has been no systematic gharial survey in the western channel of the Karnali and its tributaries (Figure 1). Historic records show that there was a resident gharial population comprising 15–20 adult gharials at Chisapani Bridge in the Karnali by the mid‐1970s which declined during bridge construction in the 1970s (Maskey 1989). No more than one gharial had been sighted in the eastern channel, and no gharial was reported from the western channel between 2016 and 2019 (Acharya et al. 2017; Bashyal et al. 2021). We recorded up to five gharials in 2024 and seven gharials in 2025 in the lower ~4 km stretch of the western channel between Sattighat bridge and the Indo‐Nepal border. Despite the availability of sand banks for basking and nesting, there are higher anthropogenic threats, including sand mining, boulder quarrying, and fishing to gharials in the upstream (Bashyal et al. 2021; this study), but anthropogenic activities are lower in the lower stretch of the Karnali close to the Indo‐Nepal border.

Interestingly, counts of gharial recorded in our surveys also coincided with the number of nests recorded in the Karnali and its tributaries, furthering the robustness of our results on gharial counts. For instance, in May and June 2024, we recorded a maximum of 8 gharials, including 6 adult females, and we recorded 5 gharial nests. Similarly, in 2025, we recorded a maximum of 12 gharials, including 10 adult females, and we recorded 11 nests (Figure 2; Table 1).

We accounted for double counting by employing a combination of techniques/methods that involved (a) conducting surveys in a single direction and in shorter duration, that is, we surveyed all rivers in consecutive days allowing shorter time windows for gharials to move within or among rivers, (b) conducting 13 population surveys that were spaced across the year including in dry seasons when water level was very low making movement of gharials within and among rivers difficult, and (c) comparing results from boat/foot surveys with the total number of nests, as nests are reliable proxies of the number of breeding females in the population (Lang et al. 2019), and gharial population in our study area is highly dominated by adult females.

Based on our snapshot assessment and conversational interviews with local residents encountered along rivers during surveys, we identified commercial‐scale sand mining and boulder quarrying and illegal fishing to be major threats to gharials in the Karnali. In tributaries of the Karnali, illegal fishing appears to be a major threat. During the course of this study, we observed the death of one adult female gharial due to being entangled in a fishing net in the Mohana.

### 4.2 Nesting and Reproduction of Gharials in the Karnali and Its Tributaries

Gharial nesting and reproduction are documented from the Rapti and the Narayani Rivers in the CNP and the Babai River in the BNP (Lang et al. 2019). Long‐term study on gharial reproduction in CNP between 2001 and 2017 showed that hatching seasons began earlier and were longer in the Rapti (June 5–June 26) and the Narayani (June 7–July 8) than in the Karnali and its tributaries (Table 1). However, the hatching season occurred simultaneously in the Karnali along its tributaries and the Babai River (June 19 in 2019 and June 17 in 2022; Bashyal et al. 2019, 2024). The hatching season of the gharial coincides with the monsoon, with rain likely acting as a natural cue (Khadka et al. 2020). Monsoon enters Nepal from the east and reaches Chitwan National Park in central Nepal earlier than the Babai and the Karnali Rivers in the west. Similarly, the pooled mean clutch size for the Karnali and its tributaries (mean = 31.81) was lower than the mean clutch size reported from the Narayani (mean = 34 ± 9.5) but slightly higher than Rapti (mean = 29.9 ± 8.2; Khadka et al. 2020) for 2001–2017. Nonetheless, the pooled mean hatching success in the Karnali and its tributaries (mean = 83.89%) was higher than the Narayani (mean = 51.6% ± 29.7%) and the Rapti (mean = 71% ± 22.9%; Khadka et al. 2020). On average, nests in the Karnali and its tributaries (mean = 5.59 ± 2.23 m) were closer to the riverbank than the Narayani (mean = 11.85 ± 8.04 m) but farther away than in the Rapti (mean = 4.46 ± 1.49 m; Khadka et al. 2020).

We recorded only two gharial hatchings in the Mohana from the 2025 batch, but none from the 2024 batch during our post‐monsoon surveys. Gharial hatchling mortality within the first 6 months is reported to be very high (70%–90% or higher) throughout its range (Lang et al. 2019). Survival rate is reported between 0.4% and 8.2% for hatchlings in the Rapti and the Narayani Rivers in the CNP in 2017–2019 (Khadka et al. 2020). No gharial hatchlings from the previous years were recorded in the Babai between 2021 and 2023 (Bashyal et al. 2024). Monsoon floods in Nepalese rivers that sweep hatchlings away are considered one of the major reasons for their extremely low survival rates (Maskey 1989; Khadka 2020). Other threats to hatchling survival include predation by certain bird species and Mugger crocodiles ( *Crocodylus palustris* ; Khadka 2020). However, empirical data are largely lacking in elucidating the exact reasons for the extremely low survival of gharial hatchlings. It should also be noted that we may not have detected hatchlings during surveys since daytime surveys such as ours are biased towards larger individuals (Bashyal et al. 2021, 2024).

During the last Red List Assessment of gharial in 2019, the gharial population in the Karnali was classified as “minor sub‐population” with no evidence of recent reproduction and recruitment (Lang et al. 2019; Bashyal et al. 2021). Our results have shown that despite a small population of gharial occurring in the Karnali and its tributaries, they are reproducing annually, with more nests being discovered than in the fully protected 46 km stretch of the Babai River in BNP, where no more than three nests have been documented annually recently (Bashyal et al. 2019, 2024).

### 4.3 Possible Seasonal Migration of Gharials in the Karnali and Its Tributaries

Findings from our surveys show that the number of gharials that occur in the Karnali and its tributaries changes seasonally as influenced by water levels and the phenology of gharials. For instance, the highest gharial count was recorded in the monsoon season (hatching season), followed by the pre‐monsoon season (nesting season), which are both characterized by high water levels.

We hypothesize two possible movement patterns of gharials in the Karnali and its tributaries. First, gharials can move among these rivers since the Patthariya and the Kaande are tributaries of the Mohana, which eventually flows into the Karnali. Gharials can move from the Karnali inner towards the Mohana and Patthariya during the monsoon to avoid flooding in the Karnali and return when the water level drops post‐monsoon. Gangetic dolphins ( *Platanista gangetica* ) also exhibit similar movement patterns in the Karnali and its tributaries (Paudel et al. 2015; Khatiwada et al. 2019).

Secondly, our findings show that the number of gharials increases during nesting and hatching seasons and decreases post‐hatching season. This observation, together with the absence of an adult male gharial in our surveys, likely suggests that adult females breed with adult male(s) in Girwa and Kaudiyala Rivers in and around Katerniaghat Wildlife Sanctuary in India and travel upstream towards the Karnali and its tributaries in Nepal for nesting. Vashistha et al. (2025) reported a total of 142 gharials, including four adult males in Katerniaghat. Although the gharial population increased from 72 in 2020 to 142 in 2024 in Katerniaghat, the number of nests decreased from 36 in 2020 to 23 in 2024, which was attributed to the reduced number of adult males (Vashistha et al. 2021, 2025). Considering the presence of adult males, albeit in small numbers, and a decrease in the number of nests despite an increase in breeding individuals in Katerniaghat, possibilities for transboundary movement of gharial, especially for mating and nesting, can't be discounted.

However, it is not possible to fully substantiate this migration pattern of gharials with our current data set. Radio telemetry studies involving adult gharials, especially the nesting females in the Karnali and its tributaries, would provide robust information on their seasonal and transboundary migration patterns.

### 4.4 Conclusion and Conservation Recommendations

We have established baseline information on the gharial population residing in the Karnali River and its tributaries by providing the first systematic accounts of their counts, nesting and reproduction, and likely seasonal and transboundary migration. There is a small resident gharial population residing in these rivers, and they are reproducing successfully and possibly connected to the gharial population in Katerniaghat Wildlife Sanctuary in India. Our results show that gharials can thrive and reproduce successfully amidst human‐dominated riverine landscapes despite a certain level of anthropogenic pressure that these rivers face, thus underscoring the role of rivers beyond protected areas for gharial conservation and the need for protecting these riverine habitats. We also identify the need for coordinated transboundary research and conservation efforts between Nepal and India to protect gharials and their riverine habitats, especially in non‐protected reaches of the river shared between the two countries. Conserving these riverine habitats will also benefit sympatrically occurring globally threatened species such as mugger crocodile, the smooth‐coated otter ( *Lutrogale perspicillata* ), and Gangetic dolphin.

Based on this study, we recommend the following conservation actions: (a) regular survey and monitoring of the Karnali and its tributaries during the breeding, nesting, and reproduction season of gharials. These surveys would be more effective if conducted in coordination with India in the Mohana River, (b) long‐term studies incorporating population and nest surveys with occupancy models will provide robust information on the status of gharials, (c) Radio/satellite tagging of female gharials, especially the nesting ones, could provide important insights into gharial movement and seasonal migration patterns, (d) regular patrolling led by the Divisional Forest Office in coordination with local communities and the Armed Police Force stationed along the Indo‐Nepal border to curb illegal fishing, and (e) conducting public outreach and educational programs in local riverine communities, including schools in the vicinities of these rivers, to aware locals aware of the importance of conserving gharials and riverine habitats.

## Author Contributions

**Ashish Bashyal:** conceptualization (lead), data curation (lead), formal analysis (lead), funding acquisition (lead), investigation (equal), methodology (equal), writing – original draft (lead), writing – review and editing (equal). **Nischal Shrestha:** data curation (equal), investigation (supporting), resources (equal), software (equal), writing – original draft (equal), writing – review and editing (equal). **Rosy Thapa:** data curation (equal), formal analysis (equal), project administration (equal), writing – original draft (equal), writing – review and editing (equal). **Bhojraj Dhungana:** investigation (equal), methodology (equal), resources (equal), writing – original draft (supporting), writing – review and editing (supporting). **Ashok Kumar Ram:** project administration (equal), resources (equal), validation (equal), writing – original draft (equal), writing – review and editing (equal). **Sabanam Pathak:** project administration (equal), resources (equal), validation (equal), writing – original draft (equal), writing – review and editing (equal). **Sandeep Shrestha:** conceptualization (equal), funding acquisition (equal), project administration (equal), supervision (lead), writing – original draft (equal), writing – review and editing (equal).

## Funding

This study was primarily supported by the Zoological Society for the Conservation of Species and Populations (Zoologische Gesellschaft für Arten‐ und Populationsschutz e.V.—ZGAP) and Zoo Heidelberg, Germany; partial support was provided by the Ocean Park Conservation Foundation, Hong Kong (Grant number: RP 01.2324) to A.B. and Biodiversity Conservancy Nepal.

## Conflicts of Interest

The authors declare no conflicts of interest.

## Data Availability

All of the data supporting the findings of this study are available within the article.
